# Distribution of advanced HIV disease from three high HIV prevalence settings in Sub-Saharan Africa: a secondary analysis data from three population-based cross-sectional surveys in Eshowe (South Africa), Ndhiwa (Kenya) and Chiradzulu (Malawi)

**DOI:** 10.1080/16549716.2019.1679472

**Published:** 2019-11-04

**Authors:** Menard L. Chihana, Helena Huerga, Gilles Van Cutsem, Tom Ellman, Eric Goemaere, Stephen Wanjala, Charlie Masiku, Elisabeth Szumilin, Jean-Francois Etard, David Maman, Mary-Ann Davies

**Affiliations:** a Epicentre, Cape Town, South Africa; b School of Public Health and Family Medicine, University of Cape Town, Cape Town, South Africa; c Epicentre, Paris, France; d MSF Southern Africa Medical Unit (SAMU), Cape Town, South Africa; e MSF Nairobi, Nairobi, Kenya; f MSF Lilongwe, Lilongwe, Malawi; g MSF, Paris, France; h International Research Unit (UMI), IRD UMI 233, INSERM U1175, Montpellier University, TransVIHMI, Montpellier, France

**Keywords:** HIV, CD4, ART, population-level, Africa

## Abstract

**Background**: Despite substantial progress in antiretroviral therapy (ART) scale up, some people living with HIV (PLHIV) continue to present with advanced HIV disease, contributing to ongoing HIV-related morbidity and mortality.

**Objective**: We aimed to quantify population-level estimates of advanced HIV from three high HIV prevalence settings in Sub-Saharan Africa.

**Methods**: Three cross-sectional surveys were conducted in (Ndhiwa (Kenya): September–November 2012), (Chiradzulu (Malawi): February–May 2013) and (Eshowe (South Africa): July–October 2013). Eligible individuals 15–59 years old who consented were interviewed at home followed by rapid HIV test and CD4 count test if tested HIV-positive. Advanced HIV was defined as CD4 < 200 cells/µl. We used logistic regression to identify patient characteristics associated with advanced HIV.

**Results**: Among 18,991 (39.2% male) individuals, 4113 (21.7%) tested HIV-positive; 385/3957 (9.7% (95% Confidence Interval [CI]: 8.8–10.7)) had advanced HIV, ranging from 7.8% (95%CI 6.4–9.5) Chiradzulu (Malawi) to 11.8% (95%CI 9.8–14.2) Ndhiwa (Kenya). The proportion of PLHIV with advanced disease was higher among men 15.3% (95% CI 13.2–17.5) than women 7.5% (95%CI 6.6–8.6) p < 0.001. Overall, 62.7% of all individuals with advanced HIV were aware of their HIV status and 40.3% were currently on ART. Overall, 65.6% of individuals not on ART had not previously been diagnosed with HIV, while only 29.6% of those on ART had been on ART for ≥6 months. Individuals with advanced HIV disease were more likely to be men (adjusted Odds Ratio [aOR]; 2.1 (95%CI 1.7–2.6), and more likely not to be on ART (aOR; 1.7 (95%CI 1.3–2.1).

**Conclusion**: In our study, about 1 in 10 PLHIV had advanced HIV with nearly 40% of them unaware of their HIV status. However, a substantial proportion of patients with advanced HIV were established on ART. Our findings suggest the need for a dual focus on alternative testing strategies to identify PLHIV earlier as well as improving ART retention.

## Background

Over the past decade, there have been substantial increases in antiretroviral therapy (ART) access for people living with HIV (PLHIV) [1]. However, a substantial number of PLHIV continue to experience advanced HIV disease, contributing to ongoing HIV-related morbidity and mortality [2]. About 940,000 HIV-related deaths occurred globally in 2017 and about 70% of these were from sub-Saharan Africa [3]. Mortality is very high among individuals with advanced HIV disease particularly those with very low CD4 count [4–7].

Rapid ART initiation improves survival of PLHIV [8,9] and most countries have now implemented the WHO recommendation to start all PLHIV on ART regardless of CD4 count, called ‘treat all’, in the hope of improving survival of PLHIV [10]. However, despite sharp increases in number of ART initiations, HIV-related mortality has declined little over recent years [11] suggesting that even in the context of ‘treat all’, specific focus has to be directed towards patients with advanced HIV disease who are at highest risk of mortality. In order to reduce high HIV-related mortality and morbidity [2,12,13], the WHO released guidelines in 2017 for managing individuals with advanced HIV disease including their rapid initiation on ART [14]. In order to apply these recommendations effectively, we need to know the number and characteristics of people with advanced HIV to plan for their management and allocate resources appropriately.

Clinic-based studies have been used to estimate the burden of advanced HIV disease [15,16]. While these studies provide a quick and relatively inexpensive way to quantify this burden, the estimates from such studies may fail to accurately reflect the true number and characteristics of individuals with advanced HIV disease, because of selection bias in clinic populations rather than at population-level.

We aimed to quantify and describe the population-level characteristics of people with advanced HIV disease using data from HIV prevalence surveys that were conducted in three sub-Saharan African countries: Ndhiwa (Kenya), Chiradzulu (Malawi) and Eshowe (South Africa).

## Methods

### Study design

We used data from three population-based cross-sectional HIV prevalence surveys conducted in Ndhiwa (Kenya: September–November 2012), Chiradzulu (Malawi: February–May 2013) and Eshowe (South Africa: July–October 2013). All the surveys were not national representative surveys. The study methods have been described in detail elsewhere [17–19]. Briefly, a two-stage sampling design was used, first using systematic sampling to select clusters based on demarcations from the national population and housing censuses, which had been conducted prior to the surveys. Then, from each selected cluster, we randomly selected 25 households Chiradzulu (Malawi) and Eshowe (South Africa); 20 households for Ndhiwa (Kenya), making the sample selection self-weighting. More households than were needed were sampled to allow these additional households to be used as replacements if no one was found at home. All individuals aged 15–59 years old who were residents of the study area or visitors who had spent the previous night in the study area were eligible for inclusion.

At the time of the survey, countries were using different ART eligibility criteria. For all countries, PLHIV were ART eligible with CD4 ≤ 350 cells/µl or WHO Stage 3 or 4 disease. Kenya prevention of mother to child transmission (PMTCT) guidelines were ‘Option A’ (ART when CD4 ≤ 350/µl for pregnant and breastfeeding women and antiretroviral prophylaxis only if maternal CD4 > 350/µl), for South Africa ‘Option B’ (ART for all pregnant and breastfeeding women until cessation of breastfeeding) and for Malawi ‘Option B+’ (lifelong ART for all pregnant and breastfeeding women).

### Data collection methods

We interviewed consenting individuals at their households using a structured questionnaire and conducted a rapid HIV test if they gave consent to be tested. For minors younger than 18 years, parental/guardian consent was sought first in Ndhiwa (Kenya) and Eshowe (South Africa) but was not required in Chiradzulu (Malawi) where minors aged 14–17 years old are considered able to give their own consent for HIV testing. Details of HIV rapid testing methods and other laboratory tests carried out including testing materials have been reported in more detail elsewhere [20]. Data were double entered using EpiData and checked for inconsistencies before analysis.

### Statistical methods and analysis

Detailed description of the demographic characteristics of the study population has been reported elsewhere [20]. For this analysis, we compared the characteristics of PLHIV with and without advanced HIV disease using chi-square tests. Since we did not have clinical data of individuals who participated in the studies, we considered all PLHIV with CD4 count <200 cells/µl as having ‘advanced HIV’ disease, with the remainder of PLHIV referred to as ‘individuals without advanced HIV’. Only data collected during the surveys were used for this analysis.

We compared the cascade of care (from diagnosis (those who knew they were HIV-positive), to those on ART, to virologically suppressed) for all individuals with advanced HIV disease stratified by country and sex using chi-square tests. As the risk of mortality and morbidity increases with decreasing CD4 cell count [4–6], we also evaluated the cascade of care only among individuals with CD4 < 100 cells/µl. Among those on ART, we further examined the distribution of advanced HIV by duration on ART, categorized as <6 months or ≥6 months on ART. We chose ≥6 months as the comparison period as WHO indicates this is long enough for a positive effect of treatment on CD4 count for those taking their medications consistently [21].

For patients on ART for ≥6 months, we compared their median period on ART between individuals with and without advanced HIV disease. We also summarized virologic suppression (<1000 copies/ml) among those on ART for ≥6 months. All estimates were weighted to account for sampling design.

We used logistic regression to assess the association between age, sex, ART status and knowing one’s HIV status and advanced HIV disease for each country and random effects logistic regression for the overall data to allow for clustering by country. Using stepwise model building, age and sex were included *a priori* and ART status was added as a main explanatory variable for advanced HIV. HIV status knowledge and viral load were not included in the final model because they were collinear with ART status. We analysed the data using STATA 15 (Stata Corporation, College Station, Texas).

### Ethics

All surveys received ethical approval from local and international ethics committees. For Ndhiwa (Kenya), local approvals were obtained from the Kenya Medical Research Institute Ethical Review Committee (KEMRI, protocol number 347), Chiradzulu (Malawi) from the National Health Sciences Research Committee (protocol number 1085) and Eshowe (South Africa) from the University of Cape Town Human Research Ethics Committee (HREC) protocol number 461/2012, and the Health Research Committee of the Health Research and Knowledge Management Unit of the Kwazulu–Natal Department of Health. International approval for all studies was obtained from the Comite´ de Protection des Personnes d’Ile de France (protocol number 12056 Ndhiwa (Kenya), 12084 Chiradulu (Malawi) and 12091 Eshowe (South Africa) [20]. All study participants were given sufficient information about the aims of the surveys and provided written consent for their inclusion in the study.

## Results

Of 21,798 eligible individuals, (6833 (31.4%) Ndhiwa (Kenya)), (8277 (38.0%) Chiradzulu (Malawi)), (6688 (30.7%) Eshowe (South Africa)), 18,991 (87.1%) participated and accepted an HIV test of which 4113 (21.7%) tested HIV-positive. Of the 2807 (12.1%) who did not participate, (757/6833 (11.1%) Ndhiwa (Kenya)), (1008/8277 (12.2%) Chiradulu (Malawi)) and (1042/6688 (15.6%) Eshowe (South Africa)), the reasons for non-participation were; 51.9% not at home, 38.6% refused, 6.1% were incapacitated and for 3.4% the reasons were unspecified. Proportion of adolescents aged 15–17 years old who refused to provide consent to participate in the study was slightly higher in Eshowe (South Africa) with 63/937 (6.7%) followed by 36/851 (4.2%) in Ndhiwa (Kenya) and 35/1066 (3.3%) in Chiradzulu (Malawi).

HIV prevalence was highest in Eshowe (South Africa), 25.2% (95% Confidence Interval [CI]: 23.6–26.9) followed by Ndhiwa (Kenya) 24.1% (95%CI 22.7–25.6) and Chiradzulu (Malawi) 17.0% (95%CI 16.0–18.1). Overall, CD4 count results were available for 3957/4113 (96.2%) individuals with 5.6% from Ndhiwa (Kenya), 4.2% Chiradzulu (Malawi) and 1.6% Eshowe (South Africa) missing CD4 count results. The majority of those missing CD4 count results were unaware of their HIV status 77.1% and not on ART 90.3%. Missing CD4 count results were mostly due to individuals refusing to provide venous blood for other tests.

Table 1 shows the distribution of baseline characteristics by HIV status. The overall distribution shows that in comparison to those without HIV, among PLHIV there was a lower proportion of HIV-positive men, youth aged 15–19 years and a higher proportion of people with no schooling, widow/ers and those doing farming as a source of income.

**Table 1.** Distribution of baseline characteristics by HIV status of 18,991 individuals who participated in the three surveys Kenya, Malawi and South Africa.

Country	Ndhiwa (Kenya)				Chiradzulu (Malawi)				Eshowe (South Africa)				All three countries			
Variable	Overall N	HIV-positive n (%)	HIV-negative n (%)	P value	Overall N	HIV-positive n (%)	HIV-negative n (%)	P value	Overall N	HIV-positive n (%)	HIV-negative n (%)	P value	Overall	HIV-positive n (%)	HIV-negative n (%)	P value
**Overall**	**6076**	**1457 (24.0)**	**4619 (76.0)**	-	**7269**	**1233 (17.0)**	**6036 (83.0)**	-	**5646**	**1423 (25.2)**	**4223 (74.8)**	-	**18991**	**4113 (21.7)**	**14878 (78.3)**	-
**Gender**																
Male	2321	457 (19.7	1864 (80.3)	<0.001	2995	394 (13.2)	2601 (86.8)	<0.001	2131	338 (15.9)	1793 (84.1)	<0.001	7447	1189 (16.0)	6258 (84.0)	<0.001
Female	3755	1000 (26.6)	2755 (73.4)		4274	839 (19.6)	3445 (80.4)		3515	1085 (30.9)	2430 (69.1)		11544	2924 (25.3)	8620 (74.7)	
**Age group**																
15–19	1260	62 (4.9)	1198 (95.1)	<0.001	1563	28 (1.8)	1535 (98.2)	<0.001	1452	70 (4.8)	1382 (95.2)		4275	160 (3.7)	4115 (96.3)	<0.001
20–34	2583	701 (27.1)	1882 (72.9)		3165	470 (14.9)	2695 (85.2)		2335	677 (29.0)	1658 (71.0)		8083	1848 (22.9)	6235 (77.1)	
35–44	1097	384 (35.0)	713 (65.0)		1392	452 (32.5)	940 (67.5)		785	386 (49.2)	399 (50.8)		3274	1222 (37.3)	2052 (62.7)	
45–59	1136	310 (27.3)	826 (72.7)		1149	283 (24.6)	866 (75.4)		1074	290 (27.0)	874 (73.0)		3359	883 (26.3)	2476 (73.7)	
**Education**																
Primary	4795	1215 (25.3)	3580 (74.7)	<0.001	4882	895 (18.3)	3987 (81.7)	<0.001	2410	609 (25.3)	1801 (74.7)	0.03	12087	2719 (22.5)	9368 (77.5)	<0.001
Secondary	922	165 (17.9)	757 (82.1)		1748	174 (10.0)	1574 (90.1)		2611	650 (24.9)		1961 (75.1)	5281	989 (18.7)	4292 (81.3)	
Tertiary	104	9 (8.7)	95 (91.4)		72	11 (15.3)	61 (84.7)		193	36 (18.7)	157 (81.4)		369	56 (15.2)	313 (84.8)	
No School	251	68 (27.1)	183 (72.9)		562	152 (27.1)	410 (73.0)		431	127 (29.5)	304 (70.5)		1244	347 (27.9)	897 (72.1)	
Missing	4	0	4		5	1	4		1	1	0		10	2	8	
**Marital status**																
Never married	1291	49 (3.8)	1242 (96.2)	<0.001	1703	48 (2.8)	1655 (97.2)	<0.001	4232	989 (23.4)	3243 (76.6)	<0.001	7226	1086 (15.0)	6140 (85.0)	<0.001
Married	4133	1095 (26.5)	3038 (73.5)		4648	859 (18.5)	3789 (81.5)		1198	345 (28.8)	853 (71.2)		9979	2299 (23.0)	7680 (77.0)	
Divorced/Separated	105	28 (26.7)	77 (73.3)		626	179 (28.6)	447 (71.4)		104	49 (47.1	55 (52.9)		835	256 (30.7)	579 (69.3)	
Widowed	511	279 (54.6)	232 (45.4)		262	141 (53.8)	121 (46.2)		107	39 (36.5)	68 (63.6)		880	459 (52.2)	421 (47.8)	
Missing	36	6	30		30	6	24		5	1	4		71	13	58	
**Occupation**																
Farming	3911	1158 (29.6)	2753 (70.4)	<0.001	3282	701 (21.4)	2581 (78.6)	<0.001	317	101 (31.9)	216 (68.1)	<0.001	7510	1960 (26.1)	5550 (73.9)	<0.001
Salaried employment	803	201 (25.0)	602 (75.0)		1803	367 (20.4)	1436 (79.7)		375	121 (32.3)	254 (67.7)		2981	689 (23.1)	2292 (76.9)	
Student/none	1288	83 (6.4)	1205 (93.6)		2136	154 (7.2)	1982 (92.8)		4689	1117 (23.8)	3572 (76.2)		8113	1354 (16.7)	6759 (83.3)	
Other	67	14 (20.9)	53 (79.1)		31	8 (25.8)	23 (74.2)		265	84 (31.7)	181 (68.3)		363	106 (29.2)	257 (70.8)	
Missing	7	1	6		17	3	14		0	0	0		24	4	20	

Overall, 385/3957 (9.7%) PLHIV had advanced HIV disease; 7.8% (95%CI 6.4–9.5) in Chiradzulu (Malawi), 9.8% (95%CI 8.0–11.9) in Eshowe (South Africa) and 11.8% (95%CI 9.8–14.2) in Ndhiwa (Kenya). The proportion of patients with advanced HIV was higher among men 15.3% (95%CI 13.2–17.5) than women 7.5% (95%CI 6.6–8.6) p < 0.001 overall and by country; 17.4% (95%CI 13.7–22.0) vs 9.3% (95%CI 7.4–11.8) in Ndhiwa (Kenya), 12.9% (95%CI 9.8–17.0) vs 5.6% (95%CI 4.2–7.4) in Chiradzulu (Malawi) and 17.0% (95%CI 12.5–22.7) vs 7.4% (95%CI 6.0–9.1) in Eshowe (South Africa). Of the individuals with advanced HIV disease, 27.0% had CD4 < 100 cells/µl and 11.7% were severely immunosuppressed (CD4 < 50 cells/µl). In all three countries, sex, ART coverage and viral load level distribution varied with CD4 level. HIV status awareness varied with CD4 level only in Chiradzulu (Malawi) and Eshowe (South Africa) (Table 2). We found no difference in proportion of patients with advanced disease by age.

**Table 2.** Distribution of baseline characteristics of 3957 HIV-positive individuals by CD4 count (CD4 < 200 vs CD4 ≥ 200 cells/µl) in the three surveys Ndhiwa (Kenya), Chiradzulu (Malawi) and Eshowe (South Africa).

Country	Ndhiwa (Kenya)				Chiradzulu (Malawi)				Eshowe (South Africa)				All three countries			
Variable	Overall N (%)	CD4 < 200 n (%)	CD4 ≥ 200 n (%)	P value	Overall N (%)	CD4 < 200 n (%)	CD4 ≥ 200 n (%)	P value	Overall N (%)	CD4 < 200 n (%)	CD4 ≥ 200 n (%)	P value	Overall N (%)	CD4 < 200 n (%)	CD4 ≥ 200 n (%)	P value
**Overall**	**1376 (100)**	**159** **(11.8)**	**1217 (88.2)**	-	**1181 (100)**	**96** **(7.8)**	**1085 (91.2)**	-	**1400** **(100)**	**130** **(9.8)**	**1270 (90.2)**	-	**3957** **(100)**	**385** **(9.7)**	**3572** **(90.3)**	-
**Gender**																
Male	426 (31.0)	72 (17.4)	354 (82.6)	<0.001	368 (31.2)	47 (12.9)	321 (87.1)	<0.001	332 (23.7)	53 (17.0)	279 (83.0)	<0.001	1126 (28.5)	172 (15.3)	954 (84.7)	<0.001
Female	950 (69.0)	87 (9.3)	863 (90.7)		813 (68.8)	49 (5.6)	764 (94.4)		1068 (72.3)	77 (7.4)	991 (92.6)		2831 (71.5)	213 (7.5)	2618 (92.5)	
**Age group**																
15–19	53 (3.9)	5 (9.4)	48 (90.6)	0.83	26 (2.2)	3 (11.5)	23 (88.5)	0.71	68 (4.9)	5 (7.4)	63 (92.6)	0.07	147 (3.7)	13 (8.8)	134 (91.2)	0.54
20–34	657 (47.8)	81 (12.3)	576 (87.7)		440 (37.3)	31 (7.1)	409 (93.0)		665 (47.5)	56 (8.4)	609 (91.6)		1762 (44.5)	168 (9.5)	1594 (90.5)	
35–44	366 (26.6)	41 (11.2)	325 (88.8)		438 (37.1)	38 (8.7)	400 (91.3)		381 (27.2)	48 (12.6)	333 (87.4)		1185 (30.0)	127 (10.7)	1058 (89.3)	
45–59	300 (21.8)	32 (10.7)	268 (89.3)		277 (23.5)	24 (8.7)	253 (91.3)		286 (20.4)	21 (7.3)	265 (92.7)		863 (21.8)	77 (8.9)	786 (91.1)	
**HIV status aware**																
Yes	850 (61.8)	93 (10.9)	757 (89.1)	0.37	916 (77.6)	61 (6.7)	855 (93.3)	<0.001	1060 (75.7)	86 (8.1)	974 (91.9)	0.01	2826 (71.6)	240 (8.5)	2586 (91.5)	<0.001
No	526 (38.2)	66 (12.6)	460 (87.5)		264 (22.4)	35 (13.3)	229 (86.7)		333 (23.8)	42 (12.6)	291 (87.4)		1123 (28.4)	143 (12.7)	980 (87.3)	
Unknown					1 (0.1)	0	1		7 (0.5)	2	5		8 (0.2)	2	6	
**ART coverage**																
On ART	581 (42.2)	51 (8.8)	530 (91.2)	0.01	763 (64.6)	44 (5.8)	719 (94.2)	<0.001	741 (52.9)	52 (7.0)	689 (93.0)	0.01	2085 (52.7)	147 (7.1)	1938 (93.0)	<0.001
Not on ART	795 (57.8)	108 (13.6)	687 86.4)		388 (32.9)	41 (10.6)	347 (89.4)		641 (45.8)	69 (10.8)	572 (89.2)		1824 (46.1)	218 (12.0)	1606 (88.1)	
Unknown					30 (2.5)	11	19		18 (1.2)	9	9		48 (1.2)	20	28	
**Viral load (copies/ml)**																
<1000	544 (39.5)	30 (5.5)	514 (94.5	<0.001	725 (61.4)	28 (3.9)	697 (96.1)	<0.001	796 (56.9)	45 (5.7)	758 (94.4)	<0.001	2065 (52.2)	103 (5.0)	1962 (95.0)	<0.001
1000+	808 (58.7)	125 (15.5)	683 (84.5)		449 (38.0)	68 (15.1)	449 (84.9)		598 (42.7)	85 (14.2)	513 (85.8)		1855 (46.9)	278 (15.0)	1577 (85.0)	
Unknown	24 (1.7)	4	20		7 (0.6)	0	7		6 (0.4)	0	6		37 (0.9)			

The cascade of care for individuals with advanced HIV disease is shown in Table 3. In Table 4, we present the cascade of care restricted to those individuals with CD4 < 100 cells/µl. Overall 240/383 (62.7%) individuals with advanced HIV disease were aware of their HIV status (two had missing HIV status awareness information), 165/365 (45.2%) had ever initiated ART (20 had missing ART status information; 11 from Chiradzulu (Malawi) and 9 from Eshowe (South Africa)), 147/365 (40.3%) were currently on ART and 103/381 (27.0%) had suppressed viral load (VL) (four had missing VL information). The cascade of care was similar for individuals with CD4 < 100 cells/µl in all three countries.

**Table 3.** Cascade of care of 385 individuals with advanced HIV disease.

Country	Ndhiwa (Kenya)				Chiradzulu (Malawi)				Eshowe (South Africa)				All three countries			
Variable	Overall N (%)	Male n (%)	Female n (%)	P value	Overall N (%)	Male n (%)	Female n (%)	P value	Overall N (%)	Male n (%)	Female n (%)	P value	Overall N (%)	Malen (%)	Female n (%)	P value
**Overall**	**159 (100)**	**72 (45.3)**	**87 (54.7)**	-	**96 (100)**	**47 (49.0)**	**49 (51.0)**	-	**130 (100)**	**53 (40.8)**	**77 (59.2)**	-	**385 (100)**	**172 (44.7)**	**213 (55.3)**	-
**HIV status awareness**																
Yes	93 (58.5)	37 (51.4)	56 (64.4)	0.10	61 (63.5)	32 (68.1)	29 (59.2)	0.37	86 (66.2)	34 (64.2)	52 (69.3)	0.54	240 (62.3)	103 (59.9)	137 (64.9)	0.31
No	66 (41.5)	35 (48.6)	31 (35.6)		35 (36.5)	15 (31.9)	20 (40.8)		42 (32.3)	19 (35.9)	23 (30.7)		143 (37.1)	69 (40.1)	74 (35.1)	
Unknown	0				0				2 (1.5)	0	2		2 (0.5)	0	2	
**Ever been on ART**																
Yes	53 (33.3)	24 (33.3)	29 (33.3)	0.99	44 (45.8)	22 (53.7)	22 (50.0)	0.73	68 (52.3)	26 (54.2)	42 (57.5)	0.72	165 (42.9)	72 (44.7)	93 (45.6)	0.87
No	106 (66.7)	48 (66.7)	58 (66.7)		41 (42.8)	19 (46.4)	22 (50.1)		53 (40.8)	22 (45.8)	31 (42.5)		200 (52.0)	89 (55.3)	111 (54.4)	
Unknown					11 (11.5)	6	5		9 (6.9)	5	4		20 (5.2)	11	9	
**Currently on ART**																
Yes	51 (32.1)	22 (30.6)	29 (33.3)	0.71	44 (45.8)	22 (53.7)	22 (50.0)	0.74	52 (40.0)	20 (41.7)	32 (43.8)	0.81	147 (38.2)	64 (39.8)	83 (40.7)	0.86
No	95 (67.9)	50 (69.4)	58 (66.7)		52 (54.2)	19 (46.3)	22 (50.0		69 (53.1)	28(58.3)	41 (56.2)		218 (56.6)	97 (60.3)	121 (59.3)	
Unknown					11 (11.5)	6	5		9 (6.9)	5	4		20 (5.2)	11	9	
**Viral load (copies/ml)**																
<1000	30 (18.8)	18 (26.1)	12 (14.0)	0.06	28 (29.2)	16 (34.0)	12 (24.5)	0.30	45 (34.6)	21 (39.6)	24 (31.2)	0.32	103 (26.8)	55 (32.5)	48 (22.6)	0.03
≥1000	125 (78.6)	51 (73.9)	74 (86.1)		68 (70.8)	31 (66.0)	37 (75.5)		85 (65.4)	32 (60.4)	53 (68.8)		278 (72.2)	114 (67.5)	164 (77.4)	
Unknown	4 (2.5)	3	1										4 (1.0)	3	1

**Table 4.** Cascade of care of 104 advanced HIV disease individuals with CD4 < 100 copies/ml (to be added to the appendix).

Country	Ndhiwa (Kenya)				Chiradzulu (Malawi)				Eshowe (South Africa)				All three countries			
Variable	Overall N (%)	Male n (%)	Female n (%)	P value	Overall N (%)	Male n (%)	Female n (%)	P value	Overall N (%)	Male n (%)	Female n (%)	P value	Overall N (%)	Male n (%)	Female n (%)	P value
**Overall**	**55 (100)**	**26 (47.3)**	**29 (52.7)**	-	**13 (100)**	**7 (53.8)**	**6 (46.2)**	-	**36 (100)**	**16 (44.4)**	**20 (55.6)**	-	**104 (100)**	**49 (47.1)**	**55 (52.9)**	-
**HIV status awareness**																
Yes	36 (65.5)	15 (57.7)	21 (72.4)	0.25	9 (69.2)	5 (71.4)	4 (66.7)	0.85	28 (77.8)	12 (75.0)	16 (80.0)	0.72	73 (70.2)	32 (65.3)	41 (74.6)	0.30
No	19 (34.6)	11 (42.3)	8 (27.6)		4 (30.8)	2 (28.6)	2 (33.3)		8 (22.2)	4 (25.0)	4 (20.0)		31 (29.8)	17 (34.7)	14 (25.5)	
**Ever on ART**																
Yes	15 (27.3)	8 (30.8)	7 (24.1)	0.58	6 (46.2)	3 (50.0)	3 (60.0)	0.74	20 (55.6)	9 (60.0)	11 (55.0)	0.77	41 (39.4)	20 (42.6)	21 (38.9)	0.71
No	40 (72.8)	18 (69.2)	22 (75.9)		5 (38.5)	3 (50.0)	2 (40.0)		15 (41.6)	6 (40.0)	10 (45.0)		60 (57.7)	27 (57.5)	33 (61.1)	
Unknown					2 (15.4)	1	1		1 (2.8)	1	0		3 (2.9)	2	2	
**Currently on ART**																
Yes	15 (27.3)	8 (30.8)	7 (24.1)	0.58	6 (46.2)	3 (50.0)	3 (60.0)	0.74	13 (36.1)	5 (33.3)	8 (40.0)	0.69	34 (32.7)	16 (34.0)	18 (33.3)	0.94
No	40 (72.7)	18 (69.2)	22 (75.9)		5 (38.5)	3 (50.0)	2 (40.0)		22 (61.1)	10 (66.7)	12 (60.0)		67 (64.4)	31 (66.0)	36 (66.7)	
Missing					2 (15.4)	1	1		1 (2.8)	1	0		3 (2.9)	2	1	
**Viral load (copies/ml)**																
<1000	9 (16.4)	5 (20.0)	4 (13.8)	0.54	3 (23.1)	2 (28.6)	1 (16.7)	0.61	9 (25.0)	6 (37.5)	3 (15.0)	0.12	21 (20.2)	13 (27.1)	8 (14.6)	0.12
≥1000	45 (81.8)	20 (80.0)	25 (86.2)		10 (76.9)	5 (71.4)	5 (83.3)		27 (75.0)	10 (62.5)	17 (85.0)		82 (78.9)	35 (72.9)	47 (85.5)	
Unknown	1 (1.8)	1	0		0	0	0		0	0	0		1 (1.0)	1	0	

**Figure 1.**
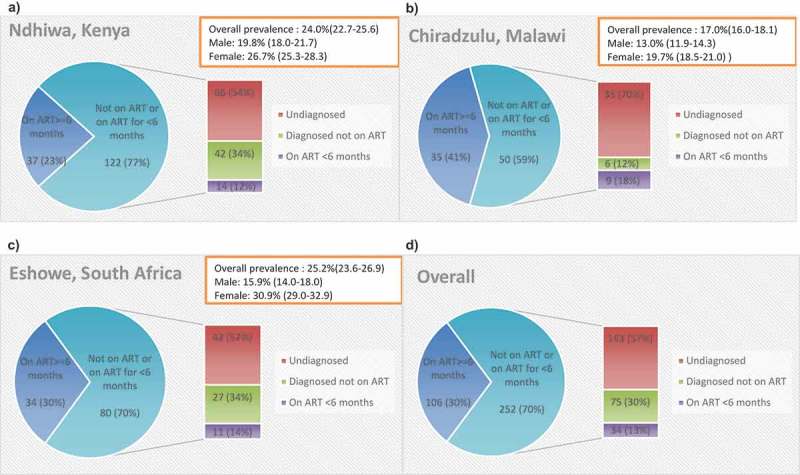
Showing distribution of 358 individuals with advanced HIV disease by ART status for each survey and overall. (a) Kenya: ART eligibility at the time of survey was CD4 ≤ 350 cells/µl or WHO Stage 3 or 4 disease and ART prophylaxis for pregnant and breast-feeding women if CD4 > 350 cells/µl (Option A)(b) Malawi: ART eligibility at the time of survey was CD4 ≤ 350 cells/µl or WHO Stage 3 or 4 disease and lifelong ART for pregnant and breastfeeding women (Option B+)(c) South Africa: ART eligibility at the time of survey was CD4 ≤ 350 cells/µl or WHO Stage 3 or 4 disease and ART for pregnant and breastfeeding women until cessation of breastfeeding (Option B).

Overall, 252/358 (70.4%) of individuals with advanced HIV disease were not on ART or were on ART for <6 months. Most of those not on ART 143/218 (65.6%) had not previously been diagnosed as HIV infected. Of those diagnosed but not on ART 13/74 (17.6%) were not linked to care (one had missing linkage to care information). We found no differences in knowledge of HIV-positivity status by age group and sex among those not on ART. Ndhiwa (Kenya) had the highest proportion of individuals not on ART or on ART for <6 months 76.7%, followed by Eshowe (South Africa) 70.2% and Chiradzulu (Malawi) 58.8% (Figure 1). We found 18/75 (24.0%) of individuals with advanced HIV disease not currently on ART had been on ART previously. Almost all were from Eshowe (South Africa) and were mostly women.

Among those who knew their HIV diagnosis with advanced HIV, the proportion who had ever been on ART ranged from 53/93 (57.0%) in Ndhiwa (Kenya), 44/61 (72.1%) Chiradzulu (Malawi) to 68/86 (79.1%) Eshowe (South Africa) and overall, the majority of them 147/165 (89.1%) were still on treatment. Most people who were still on treatment had been on ART for ≥6 months. Overall, among those who knew their HIV diagnosis with advanced HIV the proportion of men on ART for ≥6 months was lower than women (Tables 3 and 5).

**Table 5.** Distribution by time on ART for 147 individuals with advanced HIV disease on ART.

Country	Ndhiwa (Kenya)				Chiradzulu (Malawi)				Eshowe (South Africa)				All three countries			
Variable	Overall N (%)	Male n (%)	Female n (%)	P value	Overall N (%)	Male n (%)	Female n (%)	P value	Overall N (%)	Male n (%)	Female n (%)	P value	Overall N (%)	Male n (%)	Female n (%)	P value
**Time on ART**																
**Overall**	**51 (100)**	**22 (43.1)**	**29 (56.9)**	-	**44 (100)**	**22 (50.0)**	**22 (50.0)**	-	**52 (100)**	**20 (38.5)**	**32 (61.5)**	-	**147 (100)**	**64 (43.5)**	**83 (56.5)**	-
<6 months	14 (27.5)	8 (36.4)	6 (20.7)	0.21	9 (20.5)	7 (31.8)	2 (9.1)	0.06	11 (21.2)	6 (33.3)	5 (18.5)	0.26	34 (23.1)	21 (33.9)	13 (16.7)	0.02
≥6 months	37 (72.6)	14 (63.6)	23 (79.3)		35 (79.6)	15 (68.2)	20 (90.9)		34 (65.4)	12 (66.7)	22 (81.5)		106 (72.1)	41 (66.1)	65 (83.3)	
Unknown	0	0	0		0	0	0		7 (13.5)	2	5		7 (4.8)	2	5	

Of the 365/385 (94.8%) with ART status information, a further 7 were missing information about period on ART leaving 358 (93.0%) with complete ART information. Among 106/358 (29.6%) individuals with advanced HIV disease who were currently on ART for ≥6 months, the median (interquartile range (IQR)) number of months on ART was slightly lower than that of individuals without advanced HIV disease in all countries (23.1 (14.0–39.9) vs 30.4 (16.7–54.4) Ndhiwa (Kenya), 33.5 (13.8–78.1) vs 46.8 (13.0–72.2) Chiradzulu (Malawi) and 32.7 (14.2–61.9) vs 40.9 (18.9–68.6) Eshowe (South Africa)). Among all PLHIV on ART for ≥6 months, those with advanced HIV disease included fewer females (61.3% vs 74.8%) than those without advanced HIV disease (Table 6).

**Table 6.** Distribution by sex, age and viral load of 106 individuals with advanced HIV disease who had been on ART for at least 6 months.

Country	Ndhiwa (Kenya)			Chiradzulu (Malawi)			Eshowe (South Africa)		
Variable	Overall % (95% CI)	Male % (95% CI)	Female % (95% CI)	Overall % (95% CI)	Male % (95% CI)	Female % (95% CI)	Overall % (95% CI)	Male % (95% CI)	Female % (95% CI)
**Overall**	**37 (100)**	**14 (37.8)**	**23 (62.2)**	**35 (100)**	**15 (42.9)**	**20 (57.1)**	**34 (100)**	**12 (35.3)**	**22 (63.7)**
**Age group**									
15–19	2.4 (0.3–18.8)	0	4.2 (0.5–29.7)	2.6 (0.3–18.9)	0	4.8 (0.6–30.3)	0	0	0
20–34	39.4 (21.0–61.5)	26.9 (9.2–57.2)	48.8 (25.8–72.2)	15.5 (6.4–32.8)	6.0 (0.7–34.0)	23.3 (8.3–50.3)	26.2 (13.9–44.0)	0	42.3 (23.6–63.6)
35–44	34.1 (21.1–50.0)	40.0 (18.7–65.8)	29.6 (15.0–50.2)	41.4 (24.7–60.3)	38.8 (14.9–69.8)	43.4 (22.9–66.5)	46.9 (30.2–64.4)	36.7 (14.3–66.8)	53.2 (32.5–72.9)
45–59	24.1 (10.6–46.0)	33.1 (12.0–64.2)	17.4 (6.0–40.8)	40.6 (23.0–60.9)	55.2 (23.4–83.3)	28.6 (11.7–54.9)	26.8 (11.3–51.4)	63.3 (33.2–85.7)	4.5 (0.5–29.2)
**Viral load (copies/ml)**									
0–999	56.8 (35.9–75.5)	76.7 (46.0–92.7)	41.9 (20.8–66.4)	65.2 (48.8–78.6)	72.9 (51.1–87.4)	58.9 (35.8–78.6)	73.5 (52.8–87.3)	89.1 (47.8–98.7)	63.9 (41.1–81.8)
≥1000	43.2 (24.5–64.1)	23.3 (7.3–54.0)	58.1 (33.6–79.2)	34.8 (21.4–51.2)	27.1 (12.6–48.9)	41.2 (21.4–64.2)	26.6 (12.7–47.3)	10.9 (1.4–58.9)	36.1 (18.3–58.9)
All three countries									
**Overall**	**106 (100)**	**41 (38.7)**	**65 (61.3)**						
**Age group**									
15–19	1.9 (0.2–6.6)	0	3.1 (0.4–10.7)						
20–34	28.3 (20.0–37.9)	12.2 (4.1–26.2)	38.5 (26.7–51.4)						
35–44	42.5 (32.9–50.4)	41.5 (26.3–57.9)	43.1 (3.00.8–56)						
45–59	27.4 (19.1–36.9)	46.3 (30.7–62.6)	15.4 (7.6–26.5)						
**Viral load (copies/ml)**									
0–999	62.3 (52.3–71.5)	75.6 (59.7–87.6)	53.9 (41.0–66.3)						
≥1000	37.7 (28.5–47.7)	24.4 (12.3–40.3)	46.2 (33.7–59.0)						

In the multivariable analysis, those with advanced HIV disease were (adjusted Odds Ratio [aOR]); 2.1 (95%CI 1.7–2.6) times more likely to be males, and aOR; 1.7 (95%CI 1.3–2.1) times more likely to not be on ART compared to those without advanced HIV disease (Table 7).

**Table 7.** Crude and adjusted ORs for the effect of sex, age and ART on individuals with advanced HIV disease.

Country	Ndhiwa (Kenya)				Chiradzulu (Malawi)				Eshowe (South Africa)				All three countries			
Variable	N	n	Unadjusted OR (95% CI)	Adjusted OR (95% CI)	N	n	Unadjusted OR (95% CI)	Adjusted OR (95% CI)	N	n	Unadjusted OR (95% CI)	Adjusted OR (95% CI)	N	n	Unadjusted OR (95% CI)	Adjusted OR (95% CI)
**Sex**																
Male	426	72	2.0 (1.4–2.8)	2.1 (1.5–3.0)	355	41	2.2 (1.4–3.5)	2.0 (1.3–3.2)	325	48	2.3 (1.6–3.4)	2.2 (1.5–3.2)	1106	161	2.2 (1.7–2.7)	2.1 (1.7–2.6)
Female	950	87	1		795	44	1	1	1057	73	1		2802	204	1	1
**Age group**																
15–19	53	5	0.9 (0.3–2.4)	0.9 (0.3–2.6)	25	3	1.6 (0.5–5.9)	1.4 (0.4–5.1)	68	5	1.2 (0.4–3.3)	1.1 (0.4–3.1)	146	13	1.1 (0.7–2.0)	1.0 (0.5–1.9)
20–34	657	81	1.2 (0.8–1.8)	1.2 (0.8–1.9)	427	29	0.9 (0.5–1.6)	0.9 (0.5–1.6)	653	51	1.2 (0.7–2.1)	1.2 (0.7–2.1)	1737	161	1.1 (0.8–1.5)	1.1 (0.8–1.5)
35–44	366	41	1.1 (0.6–1.7)	1.0 (0.6–1.7)	426	32	1.0 (0.5–1.7)	0.9 (0.5–1.7)	378	47	2.1 (1.2–3.7)	2.1 (1.2–3.8)	1170	120	1.3 (0.9–1.7)	1.3 (0.9–1.7)
45–59	300	32	1	1	272	21	1	1	283	18	1	1	855	71	1	1
**Currently on ART**																
Yes	581	51	1		763	44	1	1	741	52	1	1	2085	147	1	1
No	795	108	1.6 (1.1–2.3)	1.6 (1.1–2.3)	387	41	1.9 (1.2–3.0)	1.8 (1.1–2.8)	641	69	1.6 (1.1–2.3)	1.7 (1.1–2.5)	1823	218	1.7 (1.4–2.2)	1.7 (1.3–2.1)
**HIV status awareness**																
Yes	850	93	1		886	50	1		1052	79	1		1120	143	1	
No	526	66	1.2 (0.8–1.6)		264	35	2.6 (1.6–4.0)		330	42	1.8 (1.2–27)		2788	222	1.6 (1.3–2.0)	
**VL (copies/ml)**																
<1000	544	30	1		722	28	1		794	45	1		2060	103	1	
≥1000	808	125	3.1 (2.1–4.7)		421	57	3.9 (2.4–6.2)		583	76	2.5 (1.7–3.7)		1812	258	3.2 (2.5–4.0)	

## Discussion

The proportion of PLHIV with advanced disease at population level ranged from 8% to 11% with men twice as likely to have advanced HIV disease as women. We found that more than half of individuals with advanced HIV disease were not on ART; most of them were not previously aware of their HIV status highlighting the need for expanded and innovative approaches to HIV testing. However, we also showed that 40% of advanced HIV patients were currently on ART. As countries move to ‘treat all’, it is likely that fewer people will experience advanced HIV. However, there may be an increasing proportion still of some PLHIV with advanced HIV disease who have a history of previously having started ART and subsequently disengaged from care; interventions to ensure retention on treatment will therefore be crucial.

To our knowledge, this is the first study to give an estimate of the prevalence of advanced HIV disease from a population-based study. Our population estimates of the prevalence of advanced HIV were generally lower than most clinical cohort-based estimates which are almost 3 times as high. A study conducted between 2013 and 2015 in Kenya [16] found that 33% of HIV-positive individuals presenting for care had advanced HIV. Another study that measured trends in CD4 count at the start of ART in 55 countries [22] found that about 37% of individuals who initiated ART in 2015 had advanced HIV overall and the proportion was even higher in some countries [15,23]. However, these higher proportions for clinic-based studies should be expected considering that many individuals present to HIV care because they are already sick and their CD4 count is measured at initiation of ART. Our population level estimates are not subject to this bias, though absence of clinical definition of advanced HIV in our survey may lead to an underestimate of true advanced HIV in the community.

Although our estimate of advanced HIV looks relatively low, this may be due to the more dynamic nature of individuals with advanced HIV disease. Individuals with advanced HIV disease are at high risk of mortality and morbidity especially if they are not started on treatment rapidly. Cross-sectional population surveys cannot estimate the longitudinal burden of advanced HIV over time as cohort studies do. Undiagnosed individuals with advanced HIV disease, however, pose a greater risk than patients in clinical care on ART, with respect to onward transmission, and also have much higher morbidity and mortality. Recent systematic reviews [5,24] looking at predictors of mortality among individuals taking ART have shown that most deaths which occurred within the first 6 months of ART initiation were associated among other factors with advanced HIV (stage 3 and 4) and low CD4 count.

While the high proportion of undiagnosed advanced HIV is a major cause of concern, another important group are those who have advanced HIV despite having initiated ART more than 6months previously, especially those that have not achieved viral suppression and those that have disengaged from care and are no longer on ART. However, our study was not designed to address this issue as we did not collect data on the cause of viral non-suppression, which could be due to inaccurate information of duration on ART, treatment failure, previous gaps in care or poor adherence. Nevertheless, we found a high proportion of individuals with advanced HIV disease who had been on ART for ≥6 months to have unsuppressed viral load. Improving access to VL testing could help identify patients who are failing on first-line treatment and facilitate earlier switch to second-line.

We found men to be twice as likely to have advanced HIV, reflecting the fact that men generally tend to access treatment later [25–27], have physiologically lower CD4 count than women and are slower in immune reconstitution while on ART [7,28]. The fact that the cascade of care among individuals with advanced HIV disease was similar by sex supports a partial contribution of poorer immune recovery among men to the burden of advanced HIV disease. Indeed, we found that among individuals with advanced HIV disease on ART for ≥6 months, men were more likely to be suppressed. Several studies have shown that because of sex differences in immune reconstitution of individuals on ART, men with lower CD4 took longer than women to recover and reach CD4 count levels associated with lower risk of mortality and morbidity [7,28]. Knowing CD4 count at diagnosis and subsequent time points, especially for older men, could assist in identifying those who are taking their treatment properly but have suboptimal immune recovery.

Although our results showed no difference in terms of HIV status awareness by sex and age group among individuals with advanced HIV disease not on ART, this could be due to small numbers in the youngest age group. Unrestricted analysis (all HIV-positive regardless of ART status) of HIV status awareness by age showed that those at youngest age were less likely to be aware of their HIV status. Although Chiradzulu (Malawi) had the highest ART coverage at the time of the survey, it also had the highest proportion of individuals with advanced HIV disease not on ART who did not know their HIV status, suggesting ongoing gaps in diagnostic coverage. Implementation of ‘treat all’ should, therefore, give special attention to individuals with advanced HIV disease to realize greater impact in reducing the burden of HIV.

The inclusion of population-level data from three different countries and comparison of patients with advanced HIV across three settings are strengths of our study. A number of people did not participate either due to refusal or not being at home. However, it is difficult to tell how these would affect the results as we did not collect data on non-participants. Other limitations include missing data especially of ART status and duration on ART. Accuracy of date of ART initiation was difficult to ascertain for the calculation of duration on ART and we did not have information on whether some individuals had defaulted and were restarting treatment. Importantly, this was a secondary data analysis of studies conducted in 2013 or earlier and the prevalence and characteristics of people with advanced HIV may have changed. Nevertheless, although most countries have adopted ‘treat all’ there may be delays in implementation at the level of all facilities and our results would thus remain relevant for settings where ‘treat all’ has not yet been fully implemented [29]. Finally, as stated in the methods, the surveys were not representative at country level and subgroup analysis was not planned at the design stage analysis, therefore, our results should be interpreted with caution.

## Conclusion

Our study demonstrates firstly that there is a substantial proportion of people with advanced HIV unaware of their status, indicating that there may be a role for different testing strategies to reach this group. Example of strategies includes HIV oral self-testing which WHO recommends [30], community-based testing strategies especially for men [31], HIV testing promotion in schools for the youngest generation and door-to-door testing campaigns. In addition, critical to reducing mortality and morbidity among individuals with advanced HIV disease is to identify them early among those newly tested HIV-positive using CD4 count test and to effectively and rapidly link them to care [14]. Although implementation of ‘treat all’ has rendered CD4 testing less important for determining ART eligibility [32], continued use of CD4 cell count test to identify individuals with advanced HIV disease ‘high risk’ will be important. Secondly, a high proportion of individuals with advanced HIV disease had previously initiated ART, suggesting the need for improved retention and outcomes after ART start, through initiatives such as using more effective first-line regimens, access to viral load testing to identify treatment failure and peer-support to encourage re-engagement in ART care in those who have become lost to follow up.

## Paper context

Despite substantial progress in antiretroviral therapy scale up, a number of people living with HIV continue to experience advanced HIV disease. Most available estimates of advanced HIV are based on clinical cohort data which is prone to selection bias. This may be the first study to quantify advanced HIV at population level. Our results suggest the need for alternative testing strategies to identify people living with HIV earlier, improving retention and virologic suppression on ART.
